# High strength extrafine pMDI beclometasone/formoterol (200/6 μg) is effective in asthma patients not adequately controlled on medium-high dose of inhaled corticosteroids

**DOI:** 10.1186/s12890-016-0335-9

**Published:** 2016-12-09

**Authors:** Pierluigi Paggiaro, Massimo Corradi, Manuela Latorre, Helene Raptis, Annamaria Muraro, Christian Gessner, Zenon Siergiejko, Mario Scuri, Stefano Petruzzelli

**Affiliations:** 1 Cardio-Thoracic and Vascular Department, University of Pisa, Ospedale di Cisanello, Via Paradisa 2, 56124 Pisa, Italy; 2 Department of Clinical and Experimental Medicine, University of Parma, Parma, Italy; 3 Chiesi Farmaceutici, Parma, Italy; 4 Department of Respiratory Medicine, University of Leipzig, Germany & POIS Leipzig GbR, Leipzig, Germany; 5 Respiratory System Diagnostics and Bronchoscopy Department, Medical University of Bialystok, Bialystok, Poland

## Abstract

**Background:**

A high strength of beclomethasone/formoterol fumarate (BDP/FF) in *a* pressurised metered dose inhaler (pMDI), which contains extrafine BDP (200 μg/actuation) and FF (6 μg/actuation) has been developed to treat those asthmatics who are not adequately controlled on previous treatments.

**Methods:**

A 12-week, randomized, double-blind, parallel group study was performed to compare the efficacy and safety of pMDI BDP/FF 200/6 (two actuations bid) with BDP 100 μg (four actuation bid) in a population of 376 randomized adult asthmatics not adequately controlled with high dose of inhaled corticosteroids (ICS) or medium dose of ICS plus long acting β_2_agonists (LABA).

**Results:**

The primary endpoint [change from baseline over the entire treatment period in average pre-dose morning peak expiratory flow (PEF)] demonstrated the superiority of BDP/FF over BDP monotherapy, with an adjusted mean difference of 19 L/min, which is above the minimal important clinical difference reported for this parameter. Overall, BDP/FF and BDP showed a similar improvement of symptom-based parameters and of the use of rescue medication after 3-month treatment. The safety profile of the two drugs was comparable, although BDP monotherapy, but not BDP/FF, slightly reduced the levels of serum cortisol.

**Conclusions:**

The study proved that pMDI BDP/FF 200/6 μg was superior to BDP alone in improving lung function with comparable safety profiles. Therefore it may be considered as an effective treatment for adults with asthma not adequately controlled with high dose of ICS monotherapy or medium dose of ICS/LABA combinations.

**Trial registration:**

ClinicalTrials.gov: NCT01577082, date 06/04/2012.

**Electronic supplementary material:**

The online version of this article (doi:10.1186/s12890-016-0335-9) contains supplementary material, which is available to authorized users.

## Background

In spite of the availability of effective treatments, many people with asthma continue to suffer from significant symptoms and impaired lung function and not well controlled asthma remains a significant social and economic burden [1, 2]. International guidelines recommend a step-wise approach to treat asthma, which is linked to increasing severity of the condition and the need to achieve disease control. According to this approach, if asthma is not adequately controlled on current therapies, treatment should be stepped up until control is achieved. The preferred treatment at step 4 is to combine a medium/high dose of ICS + LABA when control cannot be achieved with a medium dose of ICS combined with LABA, or with a high dose of ICS (http://www.ginasthma.com/).

A pressurised metered dose inhaler (pMDI) extrafine formulation containing a fixed combination of 100 μg BDP and 6 μg FF is currently available for the treatment of asthma where use of an ICS/LABA combination product is appropriate. With this combination, the usual recommended regimens are one or two inhalations twice daily [thus providing a medium total daily dose of ICS, according to The Global Initiative for Asthma (GINA) guidelines], and this schedule has been demonstrated as effective as other ICS/LABA combinations in improving symptoms and pulmonary function in asthmatics not adequately controlled with ICS monotherapy [3, 4] or in maintaining asthma control in patients already treated with ICS/LABA combination [5].

In the effort to provide caregivers more flexibility to adapt treatments to specific patients’ condition, a high strength of extrafine pMDI BDP/FF containing a higher dose of BDP (200 μg/actuation) and the same dose of FF (6 μg/actuation) has been developed. This new fixed dose combination, like the medium strength (100/6 μg) is characterized by an extrafine [i.e. mean mass aerodynamic diameter (MMAD) 1.5 μm] formulation of both active ingredients. The dosing schedule of pMDI BDP/FF 200/6 μg is 2 actuations twice daily, for a total daily dose of 800 and 24 μg for BDP and FF, respectively.

The aim of the present study was to compare the efficacy and safety of BDP/FF 200/6 μg at a daily dose of 800/24 μg with that of BDP monotherapy at a daily dose of 800 μg in asthmatic patients not adequately controlled on high dose of ICS or medium dose of ICS in ICS/LABA combinations.

## Methods

### Patients

Asthmatics aged ≥18 years, with a forced expiratory volume in the first second (FEV_1_) ≥40 and <80% of predicted normal value and with a documented positive reversibility test, defined as change in FEV_1_ ≥ 12% and ≥200 mL over baseline within 30 min after administration of 400 μg of salbutamol, were enrolled.

All patients had to be partly or not controlled (based on GINA asthma control parameters) and with an asthma control questionnaire (ACQ) > 0.75, despite previous treatment with high dose of ICS in monotherapy (BDP non-extrafine >1000 μg/day, or equivalent) or medium dose of ICS (BDP non-extrafine 500–1000 μg/day or equivalent) in combination with LABA. Patients were eligible for randomization if their asthma was still not fully controlled at the end of a 2-week run-in period.

Patients were excluded if their asthma deteriorated, resulting in a change of asthma therapy in the 4 weeks before screening.

The study was performed in accordance with the Declaration of Helsinki and Good Clinical Practice guidelines. All institutions participating to the study were granted approval by their respective Ethics Committees and Competent Authorities (the complete list in the Additional file 1). All patients gave written informed consent for their participation in the study.

### Study design

This was a phase III, multinational, multicentre, randomised, double-blind, double-dummy, active-control, 2-arm parallel group study designed to demonstrate the superiority of BDP/FF 200/6 μg pMDI extrafine (two puffs twice a day) vs. BDP 100 μg (four puffs twice a day) in change in pre-dose morning PEF.

A total of 9 visits, 2 weeks apart from each other, were performed during the study: pre-screening visit (Visit 0), screening visit (Visit 1), randomisation visit (Visit 2) and visits at Weeks 2, 4, 6, 8, 10 and 12 (Visit 3 to 8). Screening visit was followed by a 2-week run-in period (open-label), during which the patients received extrafine BDP (800 μg/day). At the randomisation visit, patients were randomised to receive either BDP/FF (800/24 μg/day) or BDP (800 μg/day) for 12 weeks.

Patients recorded daily, pre-dose morning and evening PEF, rescue and study medication use and asthma symptom (cough, wheeze, chest tightness and breathlessness) scores (score 0–3) on their electronic peak flow meter (In2itive, Vitalograph Ltd, UK); safety variables [treatment-emergent adverse events (TEAEs), adverse drug reactions (ADRs), heart rate (HR) and blood pressure (BP)] were assessed at each study visit.

Compliance was evaluated on the basis of the information recorded daily by the patient in the electronic peak flow meter, dividing the total number of doses by the number of scheduled doses. A range 75–125% was considered as a satisfactory level of compliance.

The sponsor (Chiesi) developed the protocol, with guidance from the other academic authors. The first and second authors wrote the first draft of the manuscript. All the authors reviewed and edited the manuscript and made the decision to submit the manuscript for publication. All the authors contributed to the interpretation of the data and had access to the full data.

### Efficacy assessments

PEF measurements were performed at approximately the same time every day and before the intake of run-in or study medication; an alarm reminded the patients to perform measurements. During each measurement session, a series of messages were displayed on the device to guide the patient and ensure a high quality test as follows: i) “Blow-out faster”: if the time to PEF was >120 msec; ii) “Do not cough”: if a cough was detected in the first second of the test; iii) “Blow again”: if the test was not within 40 L/min of the best PEF (to ensure repeatability). The quality criteria were based on the American Thoracic Society/European Respiratory Society (ERS) guidelines for standardisation of spirometry [6]. At each visit, data were downloaded and reviewed by the Investigator, who verified the correct use of the device, PEF values and checked patient’s compliance.

Spirometry was performed at all clinic visits at approximately the same time of day, in accordance with the ERS recommendations [6]. All sites were provided with the same spirometer and a specific procedure for centralised spirometric reading was applied.

A moderate/severe asthma exacerbation was defined as a significant deterioration of asthma and signalled by one or more of the following: i) need for an oral/parenteral corticosteroid course; ii) unscheduled medical visit for an asthma exacerbation; iii) hospitalisation or emergency room attendance for asthma.

### Safety assessments

The safety of all treatments was assessed over the entire treatment period. Electrocardiogram (ECG), haematology and blood chemistry measurements were assessed at study entry and at weeks 12.

A subset of 15% of randomised patients underwent cortisol assessment. Serum cortisol was collected at randomization and at week 12 and the corresponding area under the curve (AUC)_0-24h_, was assessed by a central laboratory.

### Statistical analysis

The primary efficacy variable of this study was the change from baseline to the entire treatment period in average pre-dose morning PEF. Baseline was calculated as the mean of the pre-dose morning PEF measurements recorded during the run-in period.

Comparison between groups was performed using an analysis of covariance (ANCOVA) model with change from baseline to the entire treatment period in average pre-dose morning PEF as dependent variable and treatment, country and gender as factors and baseline as a covariate. The sample size was calculated to demonstrate the superiority of BDP/FF over BDP in the primary efficacy variable. Assuming a mean difference of 15 L/min between treatments and a standard deviation of 40 L/min, it was calculated that 151 evaluable patients per treatment group would assure 90% power to demonstrate the superiority, with a two-side significance level of 0.050. Estimating a non-evaluable rate of 20%, a total of about 378 patients were required to be randomised.

For all the endpoints measured repeatedly during the randomised treatment by period, the change from baseline has been analysed by a Multiple Repeated Measurement Model (MRMM) including treatment, country, gender, visit/period, treatment by visit/period interaction as fixed effects and baseline and baseline by visit/period interaction as covariates. When the endpoint was a measure of the entire treatment period, the change from baseline was analysed using the same ANCOVA analysis applied for the primary endpoint.

The primary analysis has been performed in the intention to treat (ITT) population (all randomised patients who received at least one administration of the study drug and with at least one available evaluation of efficacy after baseline) and the per-protocol (PP) population (all patients from the ITT population without any major protocol deviations), while the secondary endpoints were analysed in the ITT population only.

A post-hoc analysis of the change from baseline to each visit and over the treatment period in pre-dose morning FEV_1_ was also performed considering the reversibility before randomisation [change in FEV_1_ (mL) from pre-salbutamol] as factor.

Secondary endpoints were: evening pre-dose PEF, PEF daily variability, pre-dose clinic FEV_1_, rescue medication use, day-time and night-time symptom score, asthma control days percentage, ACQ and moderate/severe exacerbation rate. Daily PEF variability was calculated for each day using the following formula: 100x [(best PEF evening-best PEF morning)/(best PEF evening + best PEF morning)/2].

Safety variables have been descriptively analysed. Cortisol assessments have been log-transformed and the change from baseline analysed as ratio and 95% CI.

## Results

### Patient’s characteristics

**Fig. 1:**
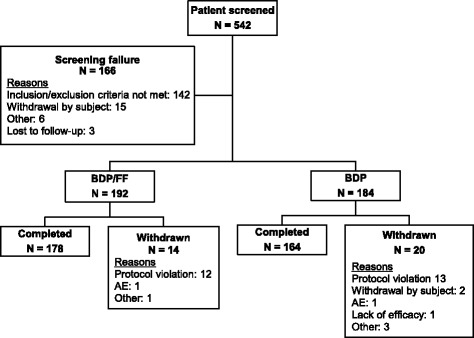
Patients’ disposition

A total of 542 patients was screened, of whom 376 were randomised to receive BDP/FF (*n* = 192) or BDP (*n* = 184). The main reason for screening failure was violation of inclusion or exclusion criteria (*n* = 142). The majority of patients completed the study (178 [92.7%] and 164 [89.1%] in the BDP/FF group and BDP group, respectively). Fourteen (7.3%) patients in the BDP/FF group and 20 (10.9%) patients in the BDP group discontinued the study mainly due to protocol violations (Fig. 1).

The demographics and clinical characteristics were similar in the two treatment groups (Table 1). In both treatment groups, there was a prevalence of females. The mean compliance to the study drug was similar in the two treatment groups was above 92%.

**Table 1:** Demographic and clinical characteristics - ITT population

		BDP/FF *N* = 184	BDP *N* = 175
Age (years)	Mean (SD)	49.5 (13.7)	49.1 (14.1)
Gender n (%)	Male	84 (45.7)	63 (36)
	Female	100 (54.3)	112 (64)
BMI (kg/m2)	Mean (SD)	26.8 (4.6)	27.1 (5.2)
Smoking n (%)	Non-smoker	151 (82.1)	147 (84)
	Ex-smoker	33 (17.9)	28 (16)
Therapy at study entry n (%)	ICS alone	16 (8.7)	15 (8.6)
	ICS/LABA	168 (91.3)	160 (91.4)
FEV1 (L)^a^	Mean (SD)	2.1 (0.6)	1.9 (0.5)
FEV1 % of pred. normal value^a^	Mean (SD)	64.7 (8.1)	64.3 (9.5)
Reversibility: FEV1 change (L)^b^	Mean (SD)	0.53 (0.3)	0.56 (0.3)
Reversibility: FEV1 % change^b^	Mean (SD)	27.7 (15.7)	30.2 (19.3)
PEF^a^	Mean (SD)	310.4 (107.6)	312.6 (102.6)
ACQ^a^	Mean (SD)	2.12 (0.6)	2.12 (0.6)

^a^Measured at randomization, ^b^Measured at screening

### Efficacy

In the ITT population pre-morning PEF (assessed during run in period) was similar in the two treatment groups [310.4 (108)] L/min in the BDP/FF group and [313 (103)] L/min in the BDP group) (Table 1).

**Fig. 2:**
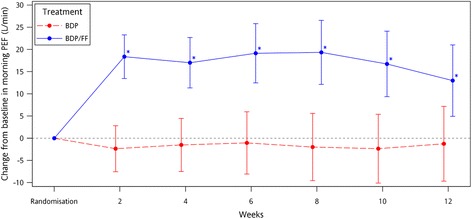
Change from baseline in average pre-dose morning PEF (L/min) [95% CI] – ITT population

In terms of adjusted mean change of PEF from baseline over the entire study, treatment period was positive in the BDP/FF group and unchanged in the BDP group (18 L/min [95% CI: 12, 24; *p* < 0.001] and -1 L/min [95% CI: -7, 5; *p* = 0.781], respectively) (Fig. 2). The difference in the adjusted mean change from baseline between the two treatment groups was statistically significant in favour of the BDP/FF group (19 L/min, 95% CI: 10, 27; *p* < 0.001), indicating superiority of BDP/FF treatment vs. BDP (Table 2). Similar differences were also observed for evening PEF (data not shown).

**Table 2:** Pre-dose morning PEF (L/min) - Change from baseline to the entire treatment period

ITT population		
	BDP/FF *N* = 184	BDP *N* = 175
Change from baseline to the entire treatment period		
n	182	170
Adjusted mean (95% CI)	17.63 (11.58, 23.68)	-0.90 (-7.26, 5.46)
Adjusted mean difference BDP/FF vs. BDP (95% CI), *p*-value	18.53 (10.33, 26.73), < 0.001	
PP population		
	BDP/FF *N* = 176	BDP *N* = 164
Change from baseline to the entire treatment period		
n	176	163
Adjusted mean (95% CI)	17.85 (11.60, 24.10),	-0.21 (-6.76, 6.35)
Adjusted mean difference BDP/FF vs. BDP (95% CI), *p*-value	18.06 (9.63, 26.49), < 0.001	

The mean daily PEF variability, at the end of run in period, was similar in BDP/FF and BDP groups (11.6 and 12.1%, respectively) and both treatments induced a reduction in PEF variability during the study, although statistically significant in BDP/FF only. In addition, the adjusted mean difference between treatment groups in daily PEF variability was statistically significant in favour of the BDP/FF group over the entire treatment period (*p* = 0.010).

**Fig. 3:**
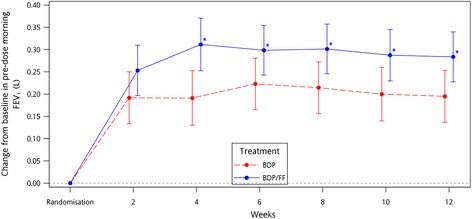
Change from baseline in pre-dose morning FEV_1_ (L) [95% CI] – Post-hoc analysis - ITT population

Both treatments showed a statistically significant increase in pre-dose morning FEV_1_ from baseline to each clinic visit and over the entire treatment period (*p* < 0.001) with a difference of 0.071 L in favour of BDP/FF group (*p* = 0.058). When reversibility test results were added as covariate (post-hoc analysis), the magnitude of the FEV_1_ difference between groups were even larger 0.087 L (*p* = 0.016) in favour of BDP/FF (Fig. 3).

The mean average use of rescue medication was similar in the BDP/FF and BDP groups at baseline (2.7 and 2.9 puffs/day respectively) and decreased significantly in both groups at the end of the treatment period (1.4 and 1.7 puffs/day respectively; *p* < 0.001 vs baseline). There were no statistically significant differences between the adjusted mean changes in use of rescue medication across treatment groups at each inter-visit period (*p*-value ranging from 0.051 to 0.586) and over the entire treatment period (*p* = 0.143). The mean number of rescue-use free days increased significantly both with BDP/FF (from 33.0 at baseline to 54.5 at end of treatment; *p* < 0.001) and BDP (from 29.4 at baseline to 50.4 at end of treatment; *p* < 0.001) with no statistically significant differences between the two groups.

The mean total day-time and night-time asthma symptom scores at baseline were similar in the BDP/FF and BDP groups (day-time asthma symptom scores: 4.03 and 4.08, respectively; night-time asthma symptom scores: 3.75 and 3.67, respectively). There were no statistically significant differences between the adjusted mean changes across treatment groups at each inter-visit period and over the entire treatment period.

The percentage of asthma symptom free days increased from 5.2 to 15.3 with BDP/FF and from 4.7 to 16.1 with BDP (*p* < 0.001 vs baseline for both groups). Similarly, the percentage of asthma control days increased from 4.6 to 15.0 with BDP/FF and from 4.4 to 15.4 with BDP (*p* < 0.001 vs baseline for both groups). No difference was observed between groups.

The percentage of asthma control days was similar in the BDP/FF and BDP groups at baseline (4.64% and 4.39%, respectively). The ACQ scores were similar in the BDP/FF and BDP at baseline (2.12 and 2.16, respectively). Both treatments resulted in a statistically significant reduction in ACQ score from baseline to Week12 (*p* < 0.001), above the minimal clinical difference of 0.5 points. The extent of the reduction was similar across treatments.

The number of patients who experienced asthma exacerbations and the corresponding number of exacerbations were slightly lower in the BDP/FF group than in the BDP group: 4 (2.2%) patients with 4 exacerbations vs. 6 (3.4%) patients with 7 exacerbations.

### Safety assessments

Overall, TEAEs were reported with similar frequency in the two treatment groups: 35 TEAEs in 29 (15.3%) patients in the CHF 1535 group and 40 TEAEs in 30 (16.7%) patients in the BDP group. Treatment-emergent ADRs were reported slightly less frequently in the CHF 1535 group than in the BDP group: 3 events in 2 (1.1%) patients vs. 5 events in 5 (2.8%) patients. Table 3 presents the ADRs reported during the study, in decreasing order of frequency.

**Table 3:** Summary of Adverse Drug Reactions (ADR)

	BDP/FF *N* = 189		BDP *N* = 180	
	Number of patients (%)	Number of events	Number of patients (%)	Number of events
Any ADR	2 (1.1)	3	5 (2.8)	5
Respiratory, thoracic and mediastinal disorders	0 (0.0)	0	3 (1.7)	3
Asthma	0 (0.0)	0	1 (0.6)	1
Dysphonia	0 (0.0)	0	1 (0.6)	1
Throat irritation	0 (0.0)	0	1 (0.6)	1
Skin and subcutaneous tissue disorders	1 (0.5)	1	1 (0.6)	1
Acne	1 (0.5)	1	0 (0.0)	0
Dermatitis contact	0 (0.0)	0	1 (0.6)	1
Gastrointestinal disorders	0 (0.0)	0	1 (0.6)	1
Nausea	0 (0.0)	0	1 (0.6)	1
General disorders and administration site conditions	1 (0.5)	1	0 (0.0)	0
Chest discomfort	1 (0.5)	1	0 (0.0)	0
Infections and infestations	1 (0.5)	1	0 (0.0)	0
Oral candidiasis	1 (0.5)	1	0 (0.0)	0

**Fig. 4:**
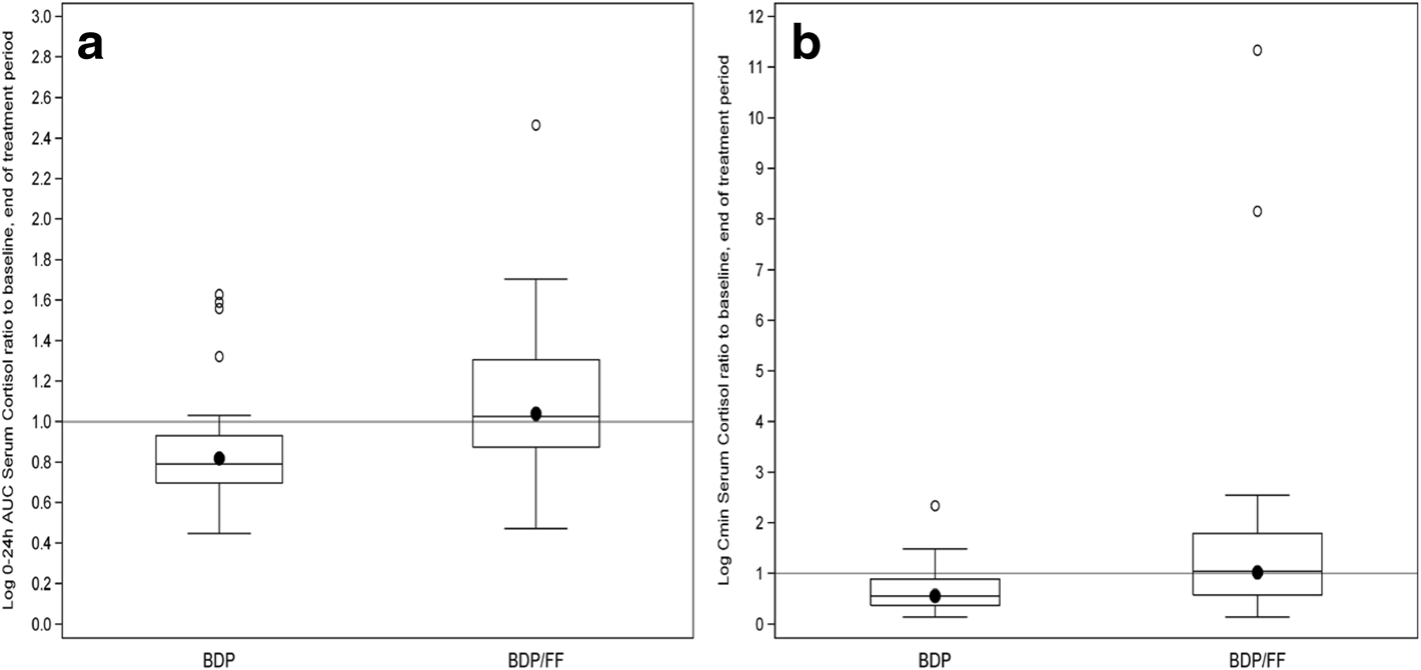
Log-transformed 24–hour serum cortisol ratio (**a**) and C_min_ serum cortisol ratio (**b**) to baseline for end of treatment period

Overall, in the BDP/FF group, serum cortisol levels remained stable from baseline to the end of the treatment period (geometric mean ratios [95% CI] for change from baseline: 1.0 [0.9, 1.2] for both AUC_0-24h_ and minimum plasma concentration (C_min_) [0.7, 1.5]), while they decreased in the BDP group (geometric mean ratios [95% CI] for change from baseline: 0.8 [0.7, 0.9] for AUC_0-24h_ and 0.6 [0.4, 0.7] for C_min_ (Fig. 4).

No clinically significant abnormalities in haematology or blood chemistry parameters were reported during the study.

With the exception of 1 patient in the BDP group, for whom the ECG reading was abnormal at the end of the treatment period, ECG readings were considered normal or not clinically significant abnormal. Overall, QTc abnormalities were infrequent and observed in a similar percentage of patients in the two treatment groups (change from screening in QTcF between 30 and 60 msec observed for 7 (3.7%) and 7 (3.9%) patients in the BDP/FF and BDP groups, respectively).

## Discussion

The primary efficacy analysis showed that BDP/FF was superior to BDP in terms of change from baseline to the entire treatment period in average pre-dose morning PEF; the target population was a group of patients with party or uncontrolled asthma despite their medium dose of ICS/LABA or high dose of ICS, which could benefit from a step up pharmacological approach. The mean difference vs. BDP was 19 L/min, which is in line with the mean value reported in the reviews of Li et al. [7] (17.9 L/min) and Cochrane [8] (19.6 L/min) and with the minimal patient perceivable improvement differences (MCID) of 18.8 L.min^-1^ which was reported by Santanello et al [9] in a similar asthma population.

The observed difference in morning PEF between BDP/FF and BDP is consistent with that observed in clinical studies when fluticasone/salmeterol high dose (500/50 μg bid) was compared to high dose of fluticasone (500 μg bid) as observed by Aubier et al [10], Boyd et al [11] and Van Noord et al [12] (22, 21 and 23 L/min, respectively). This is due to the fact that a dose of 50 μg salmeterol twice daily (as in fluticasone/salmeterol combination) is comparable to 12 μg formoterol twice daily [13, 14].

For the budesonide/formoterol (combination, the difference in morning PEF between high dose budesonide/formoterol and high dose of budesonide observed by Jenkins et al [15] and Peters et al [16] were higher than those observed in this study (33 and 34 L/min, respectively). However, the total daily dose of formoterol which is used in high strength budesonide/formoterol was 48 μg, that is twice than that used in BDP/FF and dose-response studies have shown that the degree of bronchodilation is related to the dose of inhaled FF [17]. Therefore, data from high strength budesonide/formoterol are difficult to be compared with BDP/FF and may explain the larger differences in morning PEF which have been reported by budesonide/formoterol studies. Importantly, it should be noted that in the present trial the vast majority of patients were already on a fixed dose ICS/LABA combination at screening (i.e. > 90%, see Table 1) which is significantly more than in similar studies with other combinations, and this might have reduced the room for improvement.

Overall, compared to BDP, BDP/FF resulted in a greater and statistically significant improvement of other lung function parameters: average pre-dose morning PEF (from baseline to each inter-visit period), average pre-dose evening PEF and daily PEF variability.

In addition, treatment with BDP/FF showed an improvement in FEV_1_ from baseline to each visit and throughout the treatment period compared to BDP with an increase in FEV_1_ at the end of the study period which was more than 200 ml, a cut off used to define airway reversibility [18].

There was a difference of 0.071 L in favour of BDP/FF very close to statistical significance (*p* = 0.058) and this result is in line with previous studies comparing the superiority of fixed combination vs. ICS in patients with asthma ranging in severity from mild to moderate to severe [19]. It is interesting to note that after using FEV_1_ reversibility to salbutamol as covariate in the statistical model, the change from baseline to the overall treatment period was statistically significant and the observed difference of ~0.09 L is the same as the mean value reported in the review of Lin et al [7] and also by some budesonide/formoterol data [20].

Overall, BDP/FF and BDP resulted in a similar improvement of symptom-based parameters and of the use of rescue medication. Again, these results were in part expected when looking at high dose fluticasone/salmeterol data. Aubier et al [10] showed that combination therapy and fluticasone alone all increased the mean percentage symptom-free days, symptom-free nights, rescue-free days and rescue-free nights over weeks 1–12. However, there was no significant difference between the combination and concurrent therapies for any of these measurements over 12 weeks. Our results are in line also with the data from mometasone/FF high strength. Weinstein et al. performed a study in patients who were uncontrolled with high dose of ICS [21]. At 12 weeks, the difference between mometasone 400 μg /FF 10 μg bid and mometasone 400 μg bid in ACQ was less than 0.5, thus not clinically relevant. At week 12, there were no significant between-group differences in the mean change from baseline in AQLQ(S) total score.

A possible further explanation of the similar magnitudes of asthma control between treatments may be the highly uncontrolled disease in this cohort, with the high ACQ scores.

BDP/FF and BDP showed a comparable safety profile, with no adverse events of clinical concern observed with either treatment. Overall, TEAEs and treatment-emergent ADRs were reported with low frequency in both treatment groups. In addition, all events were mild or moderate and the large majority resolved by the end of the study. Only one TEAE leading to discontinuation was reported during the study (asthma exacerbation in the BDP group which was not related to the study drug); that event was considered as not related to the study drug and resolved by the end of the study. Of note, no serious TEAEs, serious treatment-emergent ADRs or TEAEs leading to death were reported during the study.

## Conclusions

In conclusion, the study successfully demonstrated its primary objective i.e. that BDP/FF is superior to BDP alone in improving lung function in uncontrolled asthmatic patients with an acceptable safety profile. Because pulmonary function is still considered the primary outcome of all short-term studies assessing the efficacy of bronchodilator treatment in asthma, this study confirm that this new extrafine BDP/FF combination may represent a useful option in the management of moderate/severe asthma.

## Additional file

Additional file 1: List of the centres participating to the trial. (DOCX 41 kb)
