# Supplementary material for: High strength extrafine pMDI beclometasone/formoterol (200/6 μg) is effective in asthma patients not adequately controlled on medium-high dose of inhaled corticosteroids
Source: BMC Pulm Med. 2016 Dec 9;16:180. doi: 10.1186/s12890-016-0335-9 (PMC5148913; doi:10.1186/s12890-016-0335-9)
Supplement: Additional file 1: — List of the centres participating to the trial. (DOCX 41 kb) [file 12890_2016_335_MOESM1_ESM.docx]

| **COUNTRY** | **SITE** | **ETHIC COMMITTE** | **REGULATORY AUTHORITIES** |
| --- | --- | --- | --- |
| Bulgaria | All sites | Multi Center Ethics Committee  5 Sveta Nedelia, 1000 Sofia  Chairman: Dr. Anastas Stoykov | Bulgarian Drug Agency  8, Damyan Gruev str., 1303 Sofia  bda@bda.bg |
| Czech Republic | 0201  0203  0204  0205 | Ethics Committee FN Královské Vinohrady  Šrobárova 50, 100 34 Prague 10  Chairman: Prof. Dr. Jan Pachl | Státní Ústav pro Kontrolu Léčiv (SUKL)  Šrobárova 48, 100 41 PRAGUE 10  posta@sukl.cz |
|  | 0206 | Ethics Committee Hospital Mělník ul.  Pražská 528, 276 01 Mělník  Chairman: Dr. Zdenka Hradecka |  |
| France | All sites | Comité de Protection des Personnes SUD-OUEST ET OUTRE MER III  Service de Pharmacologie clinique – Groupe hospitalier Pellegrin – Bât. 1A Place Amélie Raba Léon – 33076 Bordeaux Cedex  Chairman: Prof. Jean-Pierre Duprat | Agence française de sécurité sanitaire des produits de santé (afssaps)  143-147, bd Anatole France, 93285 Saint-Denis cedex  www.afssaps.sante.fr |
| Germany | All sites | Ethics Committee State Chamber of Physicians of Saxony Schuetzenhoehe 16, D-01099 Dresden  Chairman: Prof. Dr. med. habil. Rolf Haupt | Bundesinstut für Arzneimittei und Medizinprodukte (BfArM)  Kurt Georg Kiesinger Alle 3, 53175 Bonn  www.bfarm.de |
| Hungary | All sites | Medical Research Council, Ethics Committee for Clinical Pharmacology  Arany J. u. 6-8, 1051 Budapest  Chairman: Prof. Dr. Zsuzsanna Furst | Orszàgos Gyògyszerészeti Intézet (OGYI)  Zrínyi utca 3, 1051 Budapest  www.ogyi.hu |
| Italy | All sites | Comitato Etico per la Sperimentazione Clinica dei Medicinali dell'Azienda Ospedaliero Universitaria Pisana di Pisa  Via Roma, 67 - 56126 Pisa  Chairman: Dr. Romano Danesi | Agenzia Italiana del Farmaco (AIFA)  Via del Tritone, 181 - 00187 Roma  www.agenziafarmaco.gov.it |
| Poland | All sites | Bioethical Committee of the Regional Medical Chamber in Białystok Świętojańska 7 Str., 15-082 Białystok  Chairman: Prof. Dr. hab. med. Wojciech Pędich | Urząd Rejestracji  Produktów Leczniczych, Wyrobów Medycznych i Produktów Biobójczych (CEBEK)  ul. Ząbkowska 41, 03-736 Warszawa |
| Russia | All sites | National Ethics Board under Ministry of Public Health and Social Development of the Russian Federation,  Rahmanovskiy per., 3, 127994 Moscow | Ministry of Health and Social Development of the Russian Federation  Rakhmanovsky street, 3 - 127994, Moscow |
| United Kingdom | All sites | NRES Committee London - London Bridge Research Ethics Committees (REC) Centre Charing Cross  Room 12, 4th Floor West, Charing Cross Hospital, London W6 8RF  Chairman: Prof. David Bartlett | Medicines and Healthcare products Regulatory Agency (MHRA)  151 Buckingham Palace Road, London SW1W 9SZ  www.mhra.gov.uk |
